# Supplementary material for: Design of Inhibitors That Target the Menin–Mixed-Lineage Leukemia Interaction
Source: Computation (Basel). Author manuscript; Available in PMC 2024 Jun 27. (PMC11209892; doi:10.3390/computation12010003)
Supplement: SI [file NIHMS1999806-supplement-SI.pdf]

Supplementary Information

Design of Inhibitors That Target the Menin-Mixed Lineage Leukemia Interaction

Moses N. Arthur [1,2,†], Kristeen Bebla [3,4,†], Emmanuel Broni [3,†], Carolyn Ashley [3], Miriam Velazquez [3], Xianin Hua [5], Ravi Radhakrishnan [6,7,8], Samuel K. Kwofie [9,10,*] and Whelton A. Miller III [3,4,7,*]

1 Department of Parasitology, Noguchi Memorial Institute for Medical Research (NMIMR), College of Health Sciences (CHS), University of Ghana, Legon, Accra LG 581, Ghana; marthur3@ur.rochester.edu
2 Biomedical Engineering Department, University of Rochester, Rochester, NY 14627, USA
3 Department of Medicine, Loyola University Medical Center, Loyola University Chicago, Maywood, IL 60153, USA; kbebla@luc.edu (K.B.); ebroni@luc.edu (E.B.); cashley1@luc.edu (C.A.); mvelazquez4@luc.edu (M.V.)
4 Department of Molecular Pharmacology & Neuroscience, Loyola University Medical Center, Loyola University Chicago, Maywood, IL 60153, USA
5 Department of Cancer Biology, Perelman School of Medicine, University of Pennsylvania, Philadelphia, PA 19104, USA; huax@mail.med.upenn.edu
6 Department of Bioengineering, School of Engineering and Applied Science, University of Pennsylvania, Philadelphia, PA 19104, USA; rradhak@seas.upenn.edu
7 Department of Chemical and Biomolecular Engineering, School of Engineering and Applied Science, University of Pennsylvania, Philadelphia, PA 19104, USA
8 Department of Biochemistry and Biophysics, Perelman School of Medicine, University of Pennsylvania, Philadelphia, PA 19104, USA
9 Department of Biomedical Engineering, School of Engineering Sciences, College of Basic & Applied Sciences, University of Ghana, Legon, Accra LG 77, Ghana
10 Department of Biochemistry, Cell and Molecular Biology, West African Centre for Cell Biology of Infectious Pathogens, College of Basic and Applied Sciences, University of Ghana, Accra LG 54, Ghana
* Correspondence: skkwofie@ug.edu.gh (S.K.K.); wmiller6@luc.edu (W.A.M.III)
† These authors contributed equally to this work.

Content

Figure S1: Potential energy plot of menin after energy minimization using OPLS and CHARMM36 force fields.
Figure S2: Root mean squared deviation (RMSD) [(a) and (b)] and Radius of gyration (Rg) [(c) and (d)] plots after 20 ns MD simulation of menin using OPLS and CHARMM36 force fields for: (a) and (c) full protein including disordered regions; and (b) and (d) only structured part.
Figure S3: Superimposition of the best pose from AutoDock Vina docking to the co-crystalized structures for (a) MI-2 (0RO), (b) MI-2-2 (0RT), (c) MI-89, and (d) MIV-6. For all the superimpositions, the co-crystalized ligands are in red while the docking poses are coloured yellow.
Figure S4: Protein-ligand interaction maps of menin in complex with (a) CID 71777742, (b) ZINC000103526876, (c) ZINC000085530497, (d) ZINC000095912718, (e) ZINC000070451048, (f) ZINC000085530488, (g) ZINC000095912706, (h) ZINC000103580868, and (i) ZINC000103584057.

Figure S5: Radius of gyration plots of only the structured region of the unbound menin protein and the menin-ligand complexes generated using GROMACS after 100 ns simulation. The Rg plots were generated using: (a) and (b) whole menin structure from MD run 1; (c) and (d) whole menin structure from MD run 2; (e) and (f) only structured regions of menin from MD run 2; (g) and (h) whole menin structure from MD run 3; and (i) and (j) only structured regions of menin from MD run 3. The unbound protein is coloured black while the known inhibitors CID 71777742, CID 36294 and 0RT are coloured red, green, and blue, respectively.

Figure S6: Root mean square deviation of the unbound menin protein and the menin-ligand complexes generated using GROMACS after 100 ns simulation. The RMSD plots were generated using: (a) and (b) whole menin structure from MD run 1; (c) and (d) whole menin structure from MD run 2; (e) and (f) only structured regions of menin from MD run 2; (g) and (h) whole menin structure from MD run 3; and (i) and (j) only structured regions of menin from MD run 3. The unbound protein is coloured black while the known inhibitors CID 71777742, CID 36294, and 0RT are coloured red, green and blue, respectively.

Figure S7: Root mean square fluctuation plots for menin-ligand complexes generated using GROMACS after 100 ns simulation for (a) MD run 2 and (b) MD run 3. The known inhibitors CID 71777742, CID 36294 and 0RT are coloured as purple, red, and green, respectively.

Figure S8: Per-residue energy decomposition of the menin-ligand complexes: (a) 0RT, (b) 36294, (c) 71777742, (d) ZINC000095912705, (e)ZINC000103526876, (f) ZINC000085530497, (g) ZINC000095912718, (h) ZINC000070451048, (i) ZINC000085530488, (j) ZINC000095912706, (k) ZINC000103580868, and (l) ZINC000103584057.

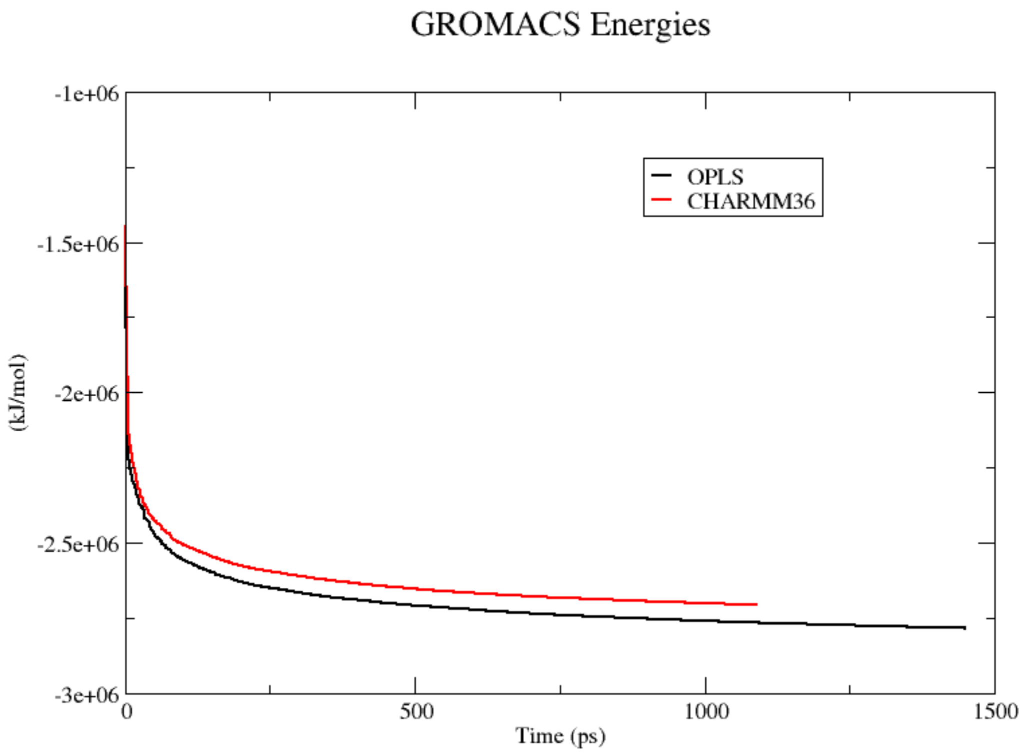

Figure S1. Potential energy plot of menin after energy minimization using OPLS and CHARMM36 force fields.

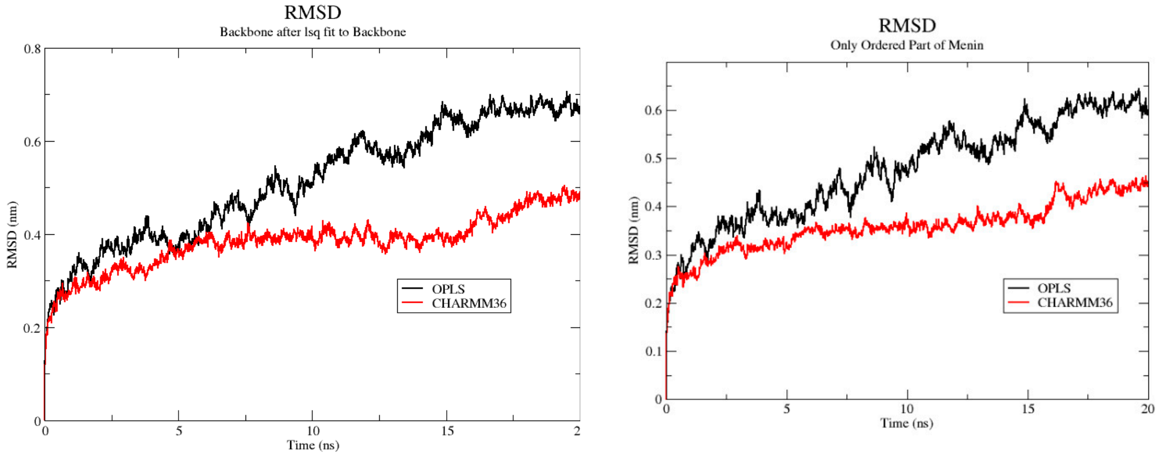

(**a**) (**b**)

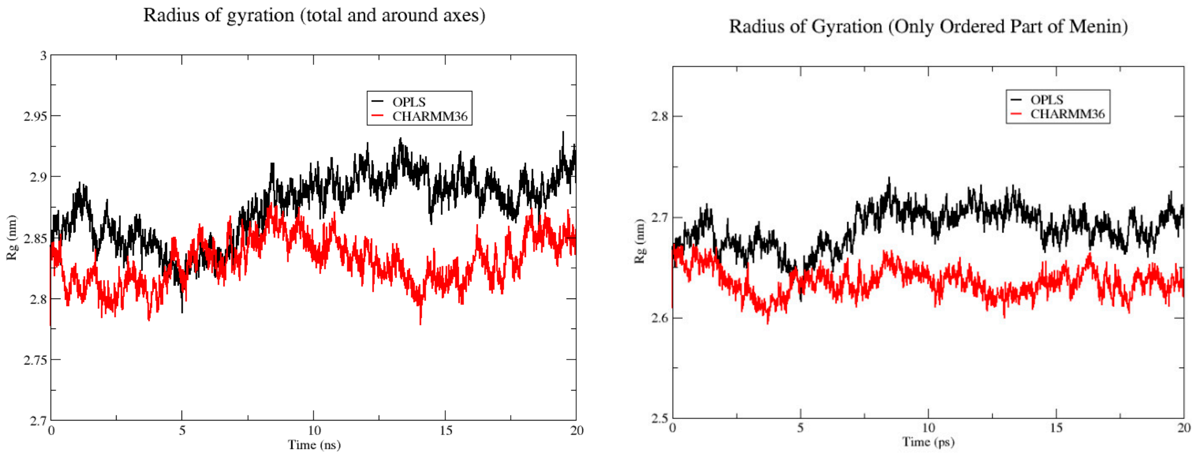

(**c**) (**d**)

Figure S2. Root mean squared deviation (RMSD) [(a) and (b)] and Radius of gyration (Rg) [(c) and (d)] plots after 20 ns MD simulation of menin using OPLS and CHARMM36 force fields for: (a) and (c) full protein including disordered regions; and (b) and (d) only structured part.

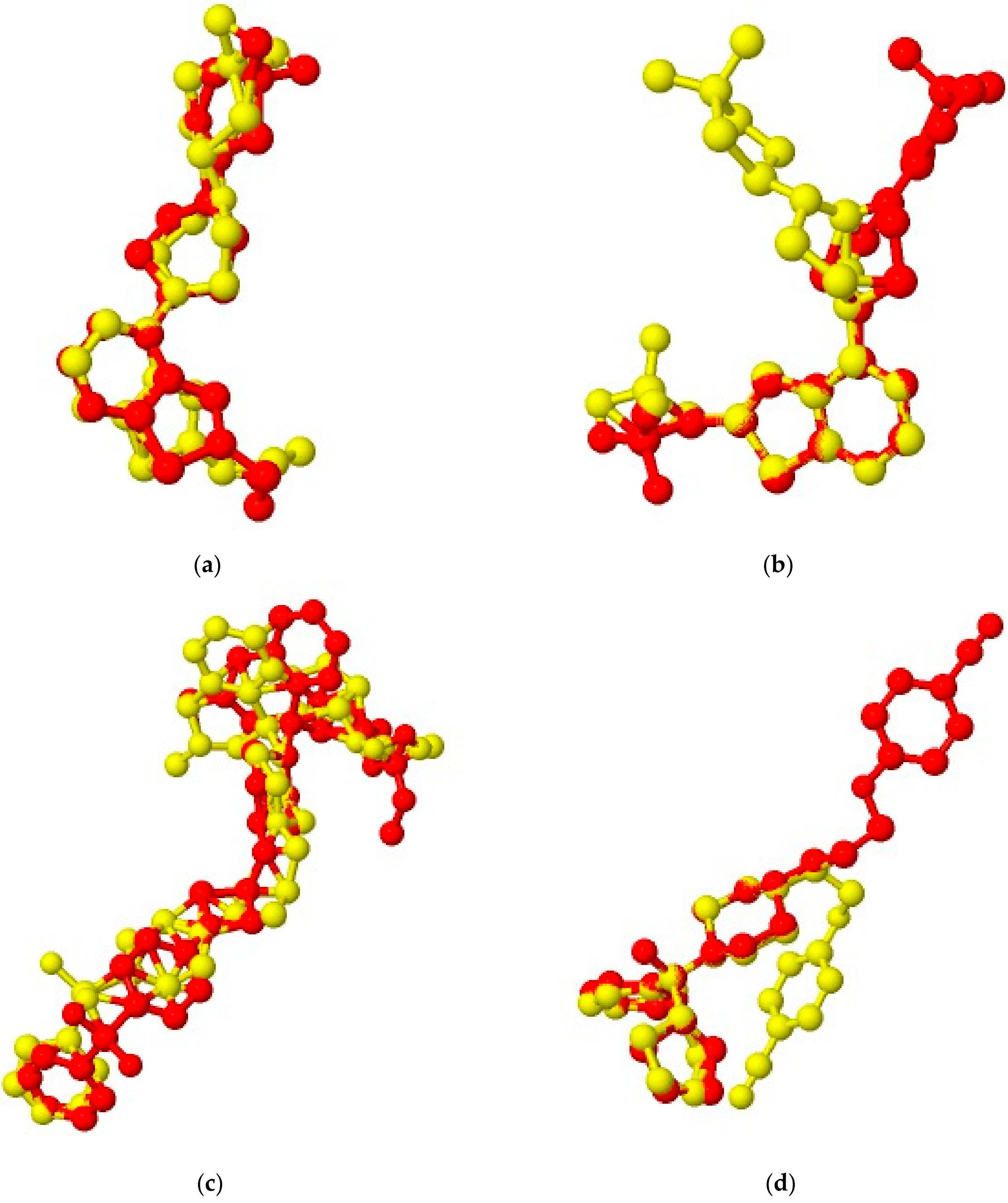

Figure S3. Superimposition of the best pose from AutoDock Vina docking to the co-crystalized structures for (a) MI-2 (0RO), (b) MI-2-2 (0RT), (c) MI-89, and (d) MIV-6. For all the superimpositions, the co-crystalized ligands are in red while the docking poses are coloured yellow.

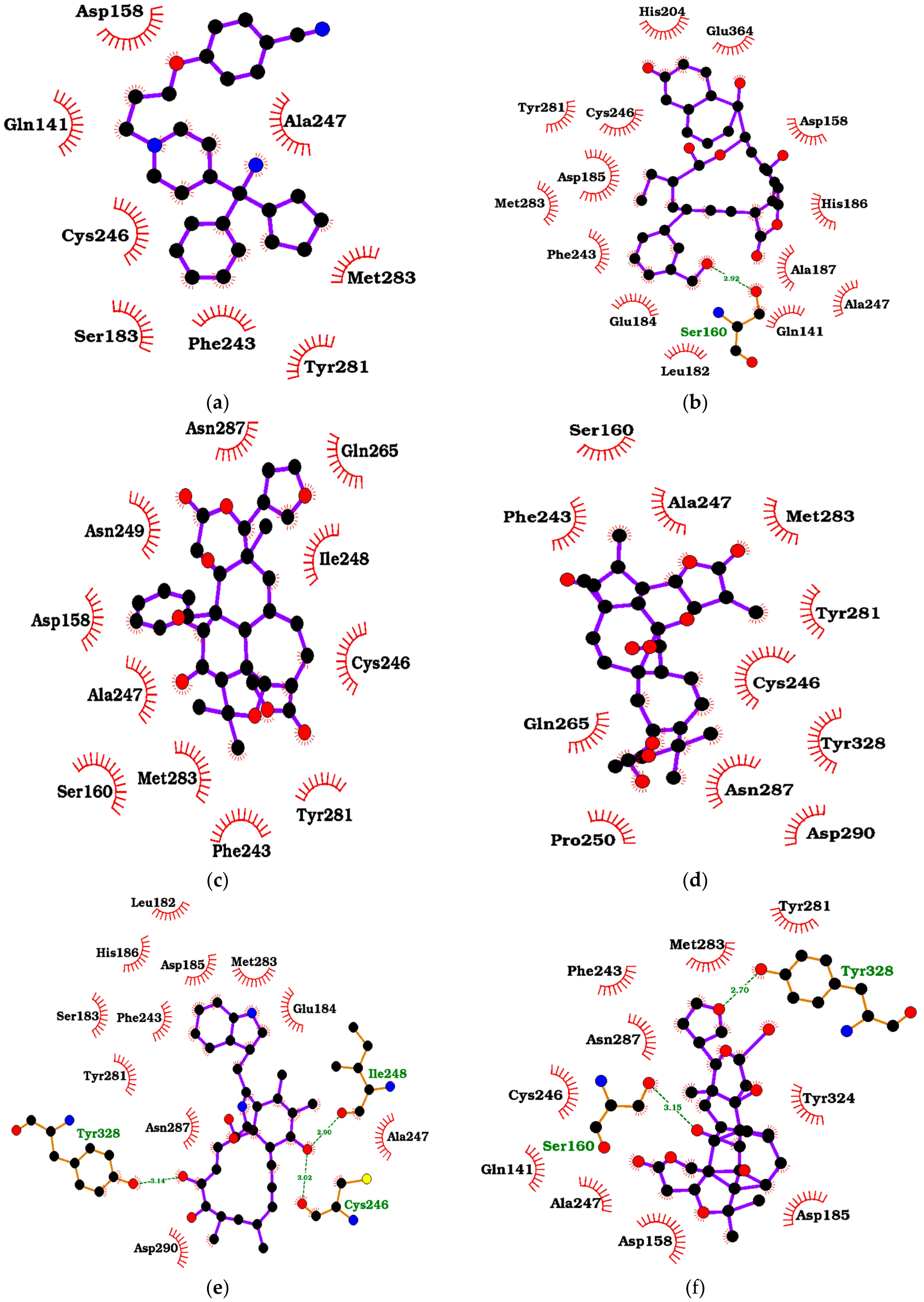

Asp158
Gln141
Ala247
Cys246
Met283
Ser183
Phe243
Tyr281
(a)
His204
Glu364
Tyr281
Cys246
Asp158
Asp185
Met283
His186
Phe243
Ala187
2.92
Ala247
Glu184
Ser160
Gln141
Leu182
(b)
Asn287
Gln265
Asn249
Ile248
Asp158
Cys246
Ala247
Ser160
Met283
Tyr281
Phe243
(c)
Ser160
Ala247
Phe243
Met283
Tyr281
Cys246
Gln265
Tyr328
Asn287
Pro250
Asp290
(d)
Leu182
His186
Asp185
Met283
Ser183
Phe243
Glu184
Tyr281
Ile248
Tyr328
Asn287
2.90
Ala247
3.14
3.02
Cys246
Asp290
(e)
Tyr281
Met283
Phe243
2.70
Tyr328
Asn287
Cys246
3.15
Tyr324
Ser160
Gln141
Ala247
Asp185
Asp158
(f)

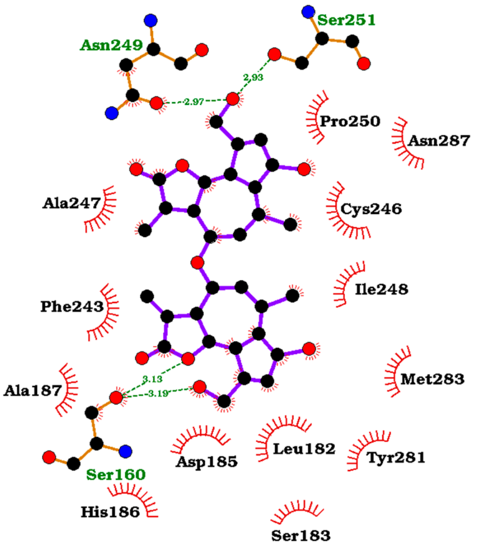

(g)

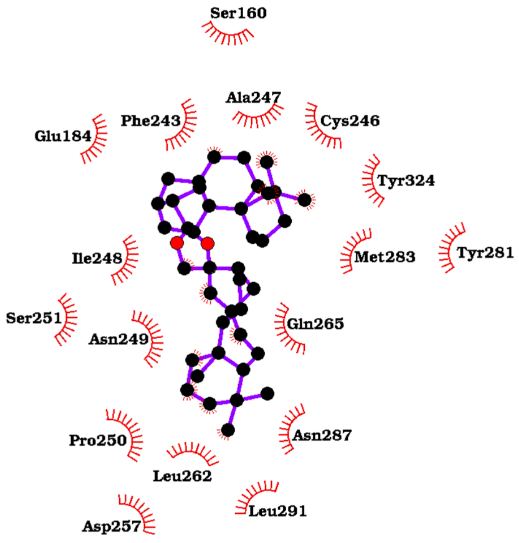

(h)

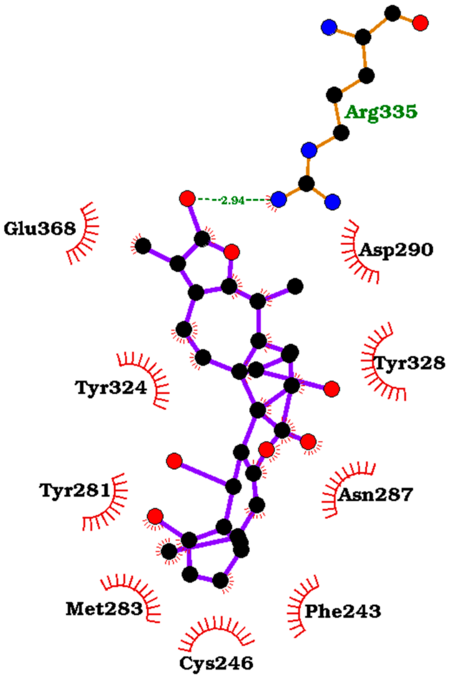

(i)

Figure S4. Protein-ligand interaction maps of menin in complex with (a) CID 71777742, (b) ZINC000103526876, (c) ZINC000085530497, (d) ZINC000095912718, (e) ZINC000070451048, (f) ZINC000085530488, (g) ZINC000095912706, (h) ZINC000103580868, and (i) ZINC000103584057.

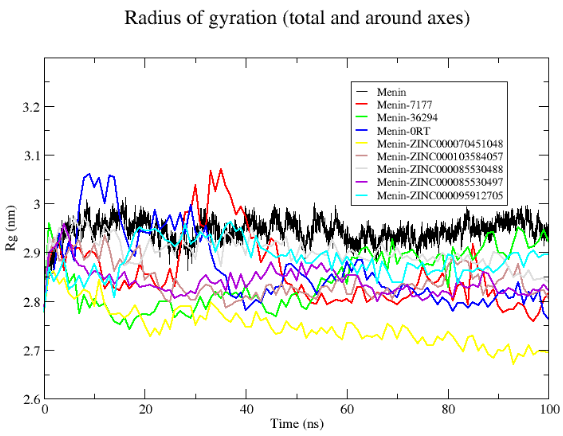
Radius of gyration (total and around axes)
Rg (nm)
Time (ns)
Menin
Menin-7177
Menin-36294
Menin-0RT
Menin-ZINC000070451048
Menin-ZINC000103584057
Menin-ZINC000085530488
Menin-ZINC000085530497
Menin-ZINC000095912705

(**a**)

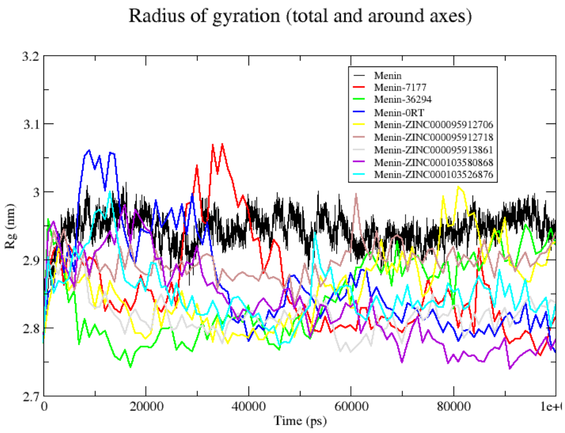
Radius of gyration (total and around axes)
Rg (nm)
Time (ps)
Menin
Menin-7177
Menin-36294
Menin-0RT
Menin-ZINC000095912706
Menin-ZINC000095912718
Menin-ZINC000095913861
Menin-ZINC000103580868
Menin-ZINC000103526876

(**b**)

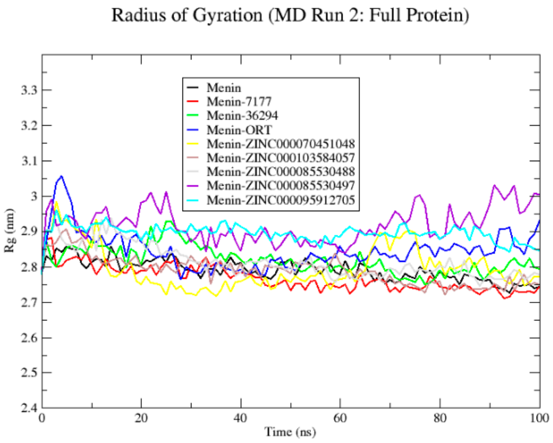
Radius of Gyration (MD Run 2: Full Protein)
Rg (nm)
Time (ns)
Menin
Menin-7177
Menin-36294
Menin-ORT
Menin-ZINC000070451048
Menin-ZINC000103584057
Menin-ZINC000085530488
Menin-ZINC000085530497
Menin-ZINC000095912705

(**c**)

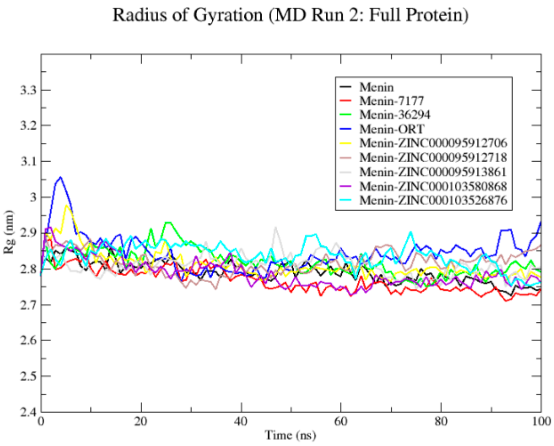
Radius of Gyration (MD Run 2: Full Protein)
Rg (nm)
Time (ns)
Menin
Menin-7177
Menin-36294
Menin-ORT
Menin-ZINC000095912706
Menin-ZINC000095912718
Menin-ZINC000095913861
Menin-ZINC000103580868
Menin-ZINC000103526876

(**d**)

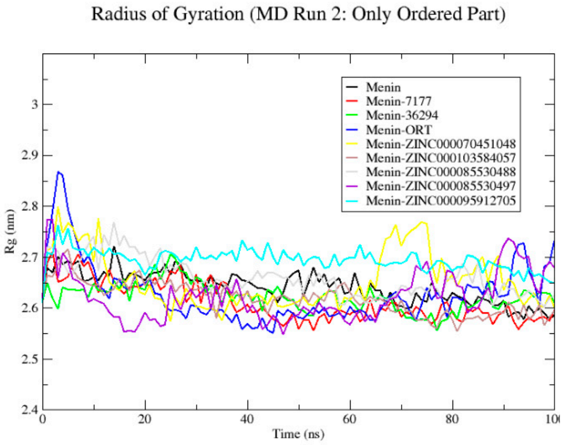
Radius of Gyration (MD Run 2: Only Ordered Part)
Rg (nm)
Time (ns)
Menin
Menin-7177
Menin-36294
Menin-ORT
Menin-ZINC000070451048
Menin-ZINC000103584057
Menin-ZINC000085530488
Menin-ZINC000085530497
Menin-ZINC000095912705

(**e**)

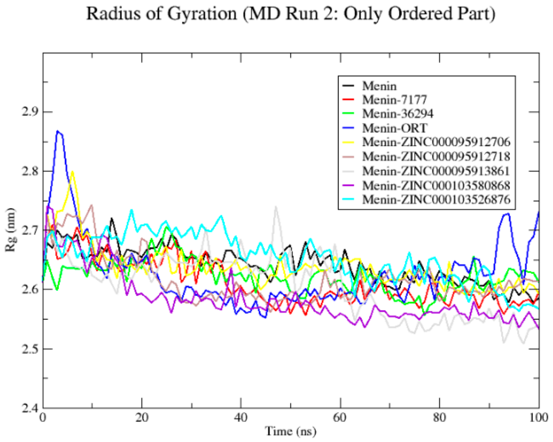
Radius of Gyration (MD Run 2: Only Ordered Part)
Rg (nm)
Time (ns)
Menin
Menin-7177
Menin-36294
Menin-ORT
Menin-ZINC000095912706
Menin-ZINC000095912718
Menin-ZINC000095913861
Menin-ZINC000103580868
Menin-ZINC000103526876

(**f**)

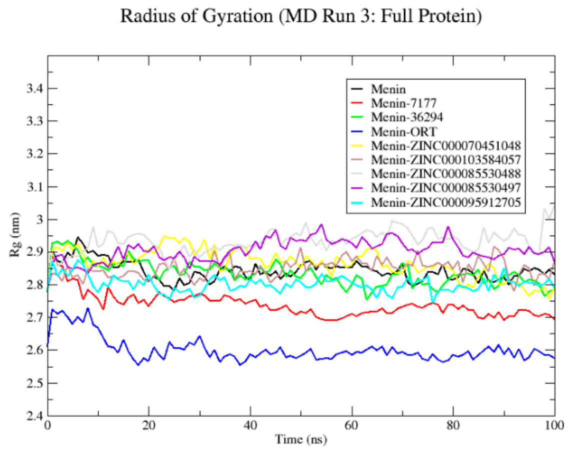

(**g**)

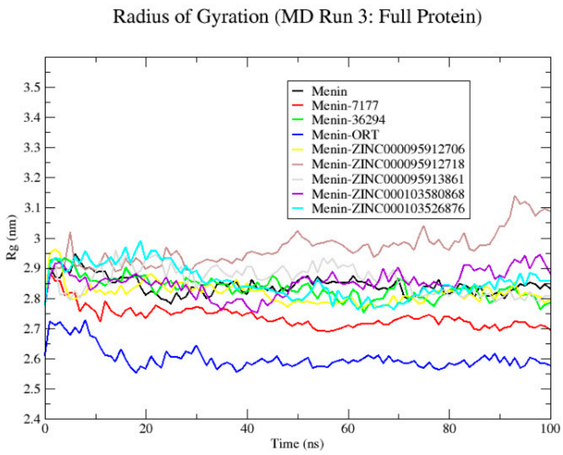

(**h**)

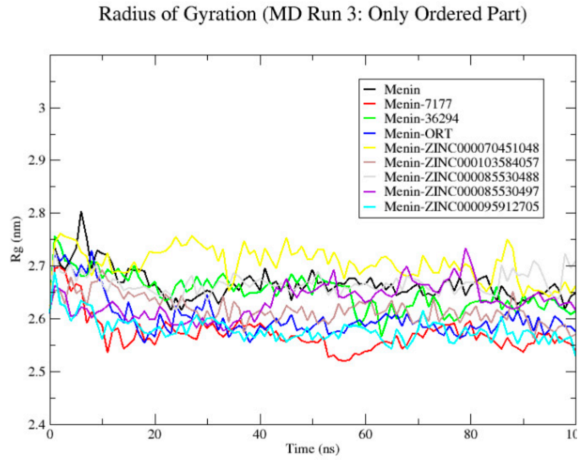

(**i**)

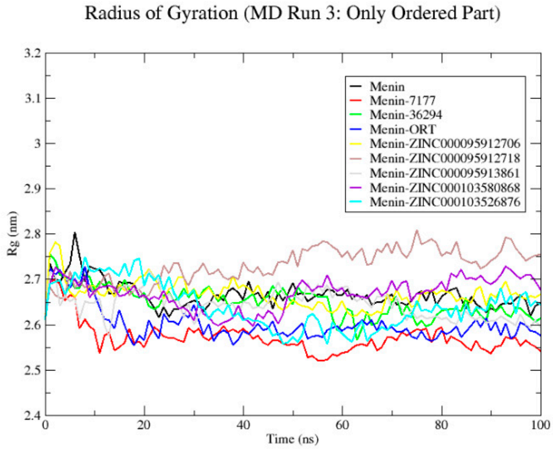

(**j**)

Figure S5. Radius of gyration plots of only the structured region of the unbound menin protein and the menin-ligand complexes generated using GROMACS after 100 ns simulation. The Rg plots were generated using: (a) and (b) whole menin structure from MD run 1; (c) and (d) whole menin structure from MD run 2; (e) and (f) only structured regions of menin from MD run 2; (g) and (h) whole menin structure from MD run 3; and (i) and (j) only structured regions of menin from MD run 3. The unbound protein is coloured black while the known inhibitors CID 71777742, CID 36294 and 0RT are coloured red, green, and blue, respectively.

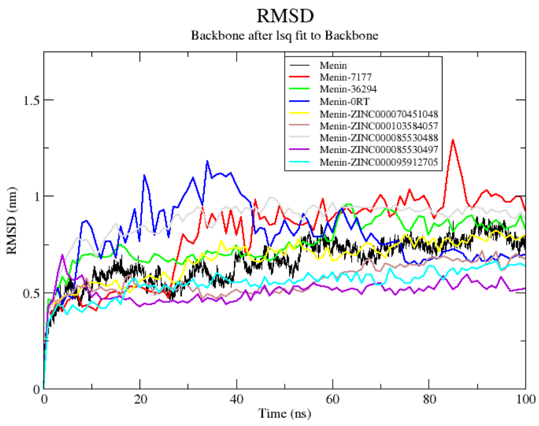
RMSD
Backbone after lsq fit to Backbone
RMSD (nm)
Time (ns)
Menin
Menin-7177
Menin-36294
Menin-0RT
Menin-ZINC000070451048
Menin-ZINC000103584057
Menin-ZINC000085530488
Menin-ZINC000085530497
Menin-ZINC000095912705

(**a**)

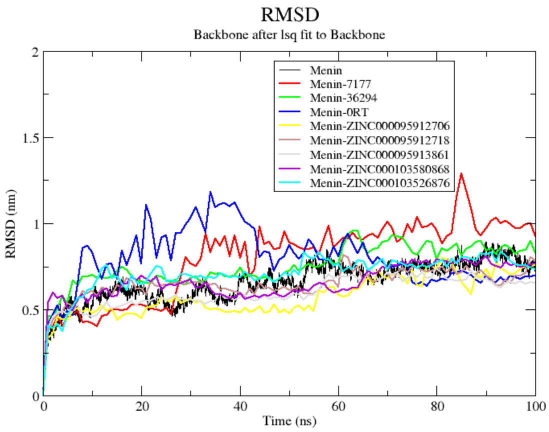
RMSD
Backbone after lsq fit to Backbone
RMSD (nm)
Time (ns)
Menin
Menin-7177
Menin-36294
Menin-0RT
Menin-ZINC000095912706
Menin-ZINC000095912718
Menin-ZINC000095913861
Menin-ZINC000103580868
Menin-ZINC000103526876

(**b**)

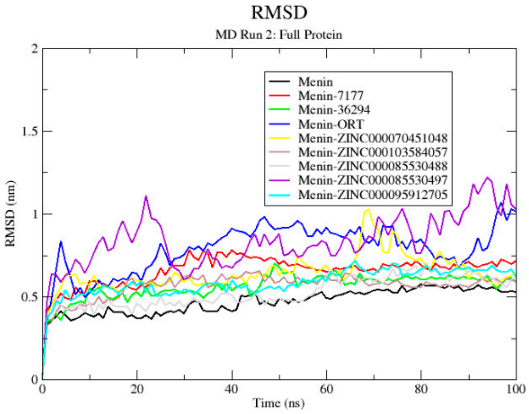
RMSD
MD Run 2: Full Protein
RMSD (nm)
Time (ns)
Menin
Menin-7177
Menin-36294
Menin-ORT
Menin-ZINC000070451048
Menin-ZINC000103584057
Menin-ZINC000085530488
Menin-ZINC000085530497
Menin-ZINC000095912705

(**c**)

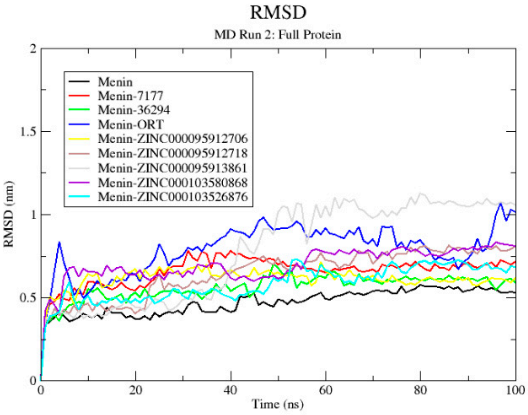
RMSD
MD Run 2: Full Protein
RMSD (nm)
Time (ns)
Menin
Menin-7177
Menin-36294
Menin-ORT
Menin-ZINC000095912706
Menin-ZINC000095912718
Menin-ZINC000095913861
Menin-ZINC000103580868
Menin-ZINC000103526876

(**d**)

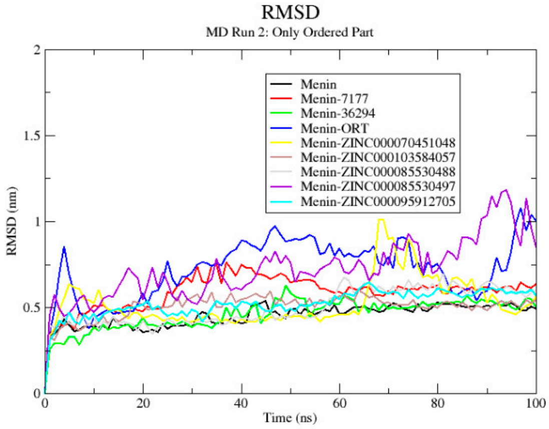
RMSD
MD Run 2: Only Ordered Part
RMSD (nm)
Time (ns)
Menin
Menin-7177
Menin-36294
Menin-ORT
Menin-ZINC000070451048
Menin-ZINC000103584057
Menin-ZINC000085530488
Menin-ZINC000085530497
Menin-ZINC000095912705

(**e**)

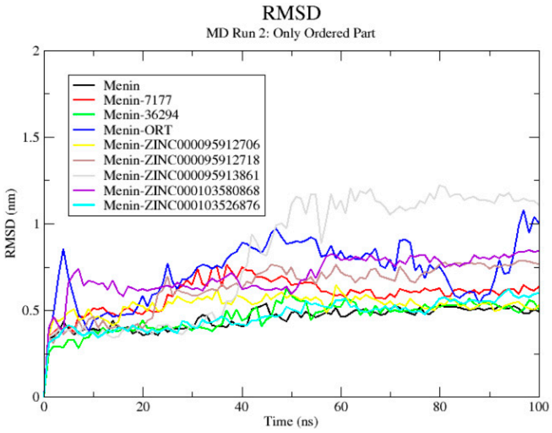
RMSD
MD Run 2: Only Ordered Part
RMSD (nm)
Time (ns)
Menin
Menin-7177
Menin-36294
Menin-ORT
Menin-ZINC000095912706
Menin-ZINC000095912718
Menin-ZINC000095913861
Menin-ZINC000103580868
Menin-ZINC000103526876

(**f**)

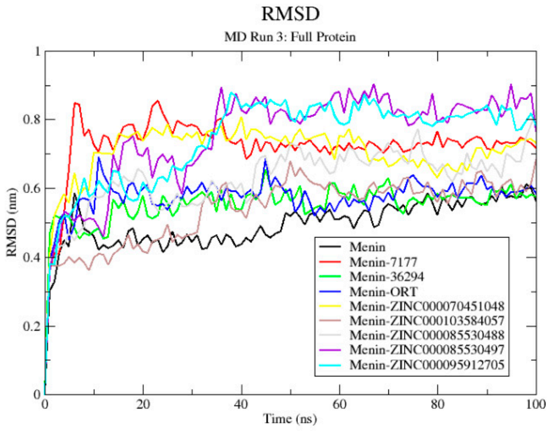

(**g**)

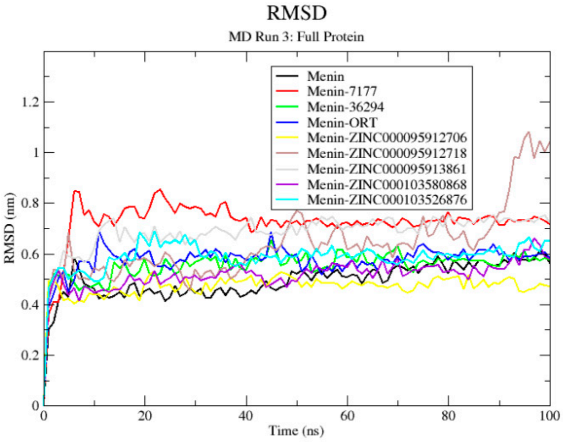

(**h**)

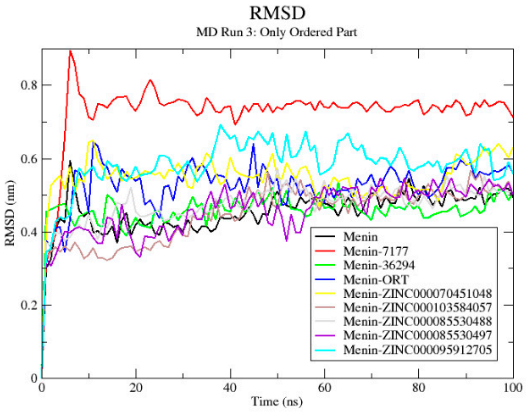

(**i**)

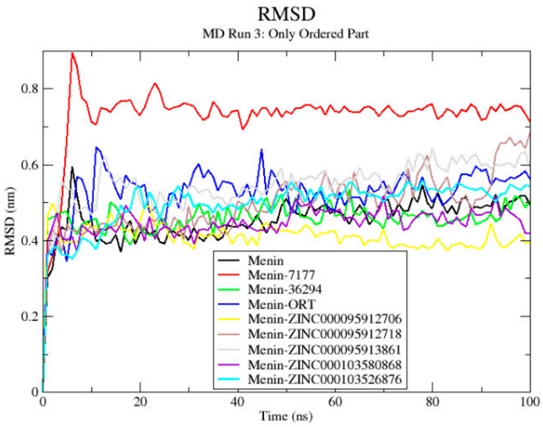

(**j**)

Figure S6. Root mean square deviation of the unbound menin protein and the menin-ligand complexes generated using GROMACS after 100 ns simulation. The RMSD plots were generated using: (a) and (b) whole menin structure from MD run 1; (c) and (d) whole menin structure from MD run 2; (e) and (f) only structured regions of menin from MD run 2; (g) and (h) whole menin structure from MD run 3; and (i) and (j) only structured regions of menin from MD run 3. The unbound protein is coloured black while the known inhibitors CID 71777742, CID 36294, and 0RT are coloured red, green and blue, respectively.

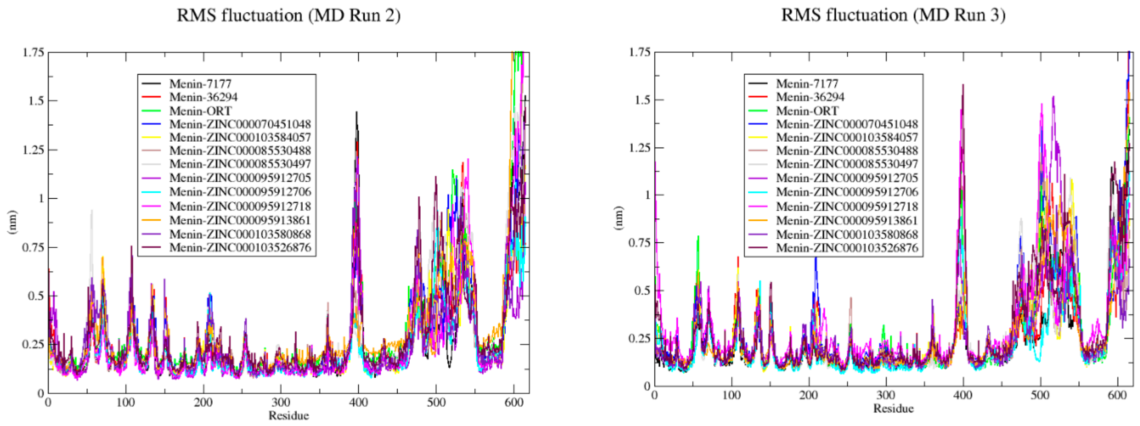

(**a**) (**b**)

Figure S7. Root mean square fluctuation plots for menin-ligand complexes generated using GROMACS after 100 ns simulation for (a) MD run 2 and (b) MD run 3. The known inhibitors CID 71777742, CID 36294 and 0RT are coloured as purple, red, and green, respectively.

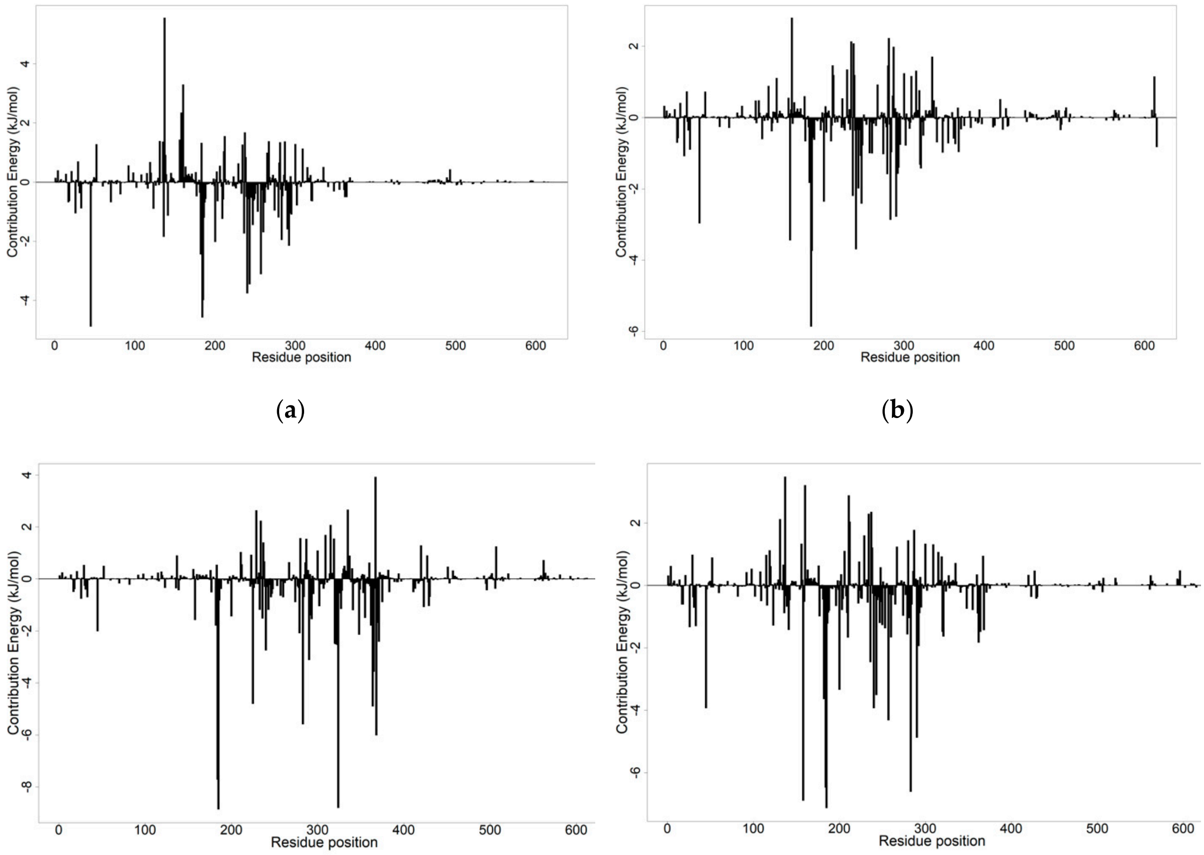

(**a**) (**b**)

(c) (d)

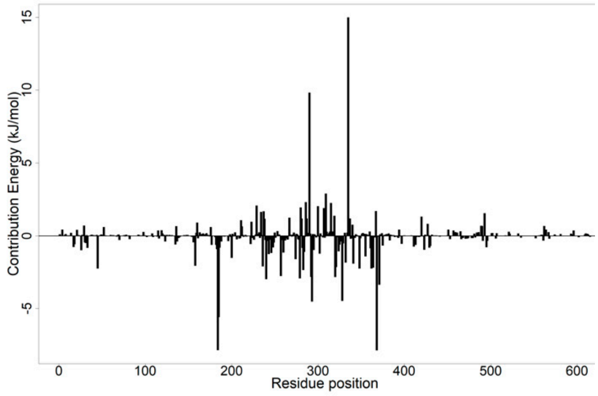
Contribution Energy (kJ/mol)
Residue position

(e)

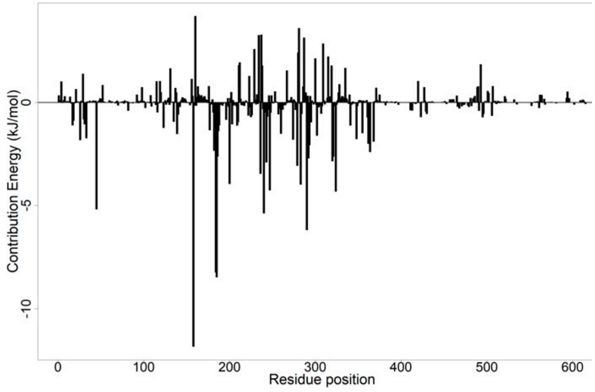
Contribution Energy (kJ/mol)
Residue position

(f)

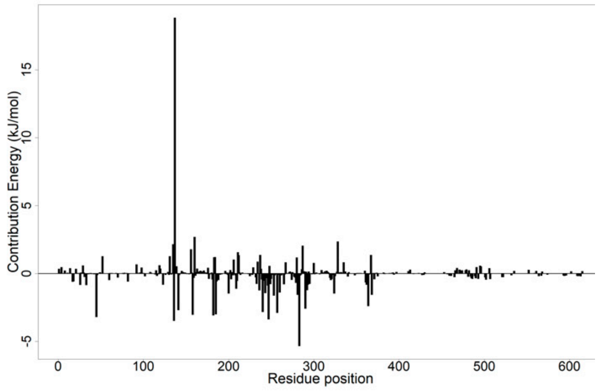
Contribution Energy (kJ/mol)
Residue position

(g)

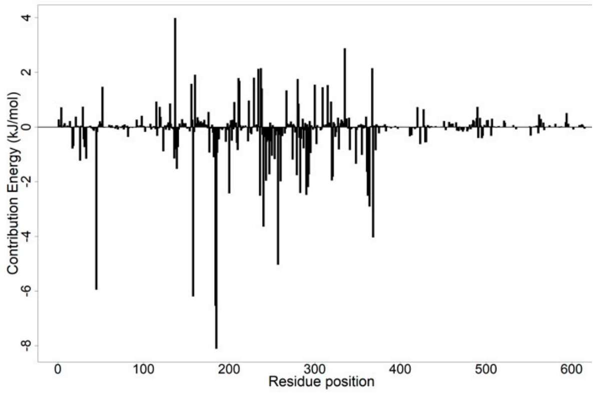
Contribution Energy (kJ/mol)
Residue position

(h)

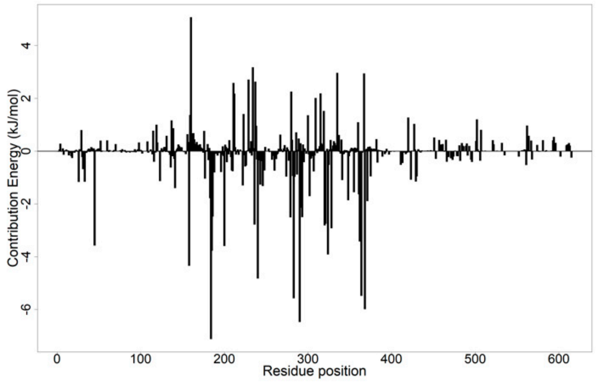
Contribution Energy (kJ/mol)
Residue position

(i)

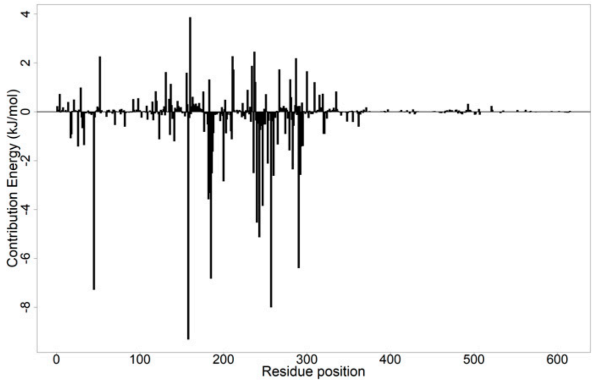
Contribution Energy (kJ/mol)
Residue position

(j)

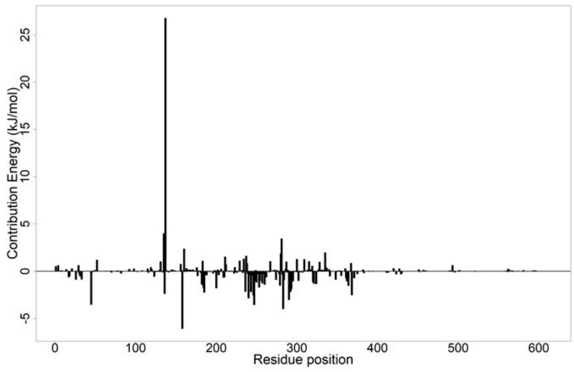

(k)

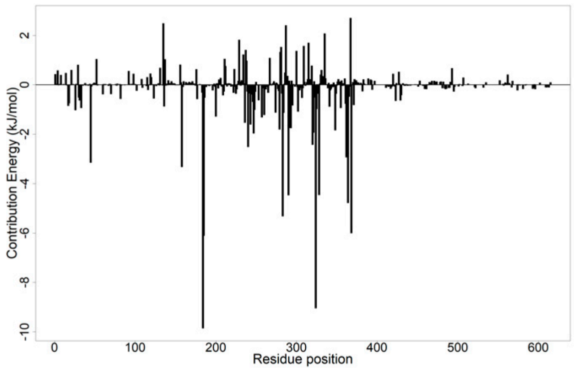

(l)

Figure S8. Per-residue energy decomposition of the menin-ligand complexes: (a) 0RT, (b) 36294, (c) 71777742, (d) ZINC000095912705, (e) ZINC000103526876, (f) ZINC000085530497, (g) ZINC000095912718, (h) ZINC000070451048, (i) ZINC000085530488, (j) ZINC000095912706, (k) ZINC000103580868, and (l) ZINC000103584057.
